# A Case Report of Acute Achilles Tendon Rupture in a Patient With Multimorbidity Treated With Endoscopic Flexor Hallucis Longus Transfer

**DOI:** 10.7759/cureus.76741

**Published:** 2025-01-01

**Authors:** Michail Kotsapas, Alexandros Eleftheropoulos, Christos Koukos, Chrysanthos Chrysanthou, Ioannis Gigis, George K Paraskevas, Nikolaos Anastasopoulos

**Affiliations:** 1 Orthopedics and Traumatology, General Hospital of Naousa, Naousa, GRC; 2 Anatomy and Surgical Anatomy, Faculty of Health Sciences, School of Medicine, Aristotle University of Thessaloniki, Thessaloniki, GRC; 3 Orthopedics, Sports Trauma and Pain Institute, Thessaloniki, GRC; 4 Orthopedics and Traumatology, Interbalkan Medical Center, Thessaloniki, GRC; 5 2nd Orthopedics, Aristotle University of Thessaloniki, Thessaloniki, GRC

**Keywords:** acute achilles tendon rupture, comorbidities, endoscopic, flexor hallucis longus transfer, high-risk patients, minimally invasive surgery, multimorbidity

## Abstract

Acute Achilles tendon rupture (AATR) in patients with multimorbidity poses a significant therapeutic challenge to surgeons because of the increased risk for wound-healing-related complications. Thus, nonoperative management has been these individuals' most widely adopted treatment. We report a case of a 66-year-old patient with AATR who was treated with endoscopic flexor hallucis longus (FHL) transfer. His medical history was remarkable for recent stroke, hypertension, prediabetes, pemphigus under oral methylprednisolone, smoking, and recent pneumonia. The patient was evaluated up to two years postoperatively and was satisfied with the outcome since he was able to maintain his pre-traumatic activity level. No complications were noted. The Achilles tendon total rupture score was 92 out of a maximum of 100. This favorable outcome indicates that endoscopic FHL transfer may be a safe alternative treatment option for patients with an increased risk of surgical complications.

## Introduction

Acute Achilles tendon rupture (AATR) is a common injury that has become more prevalent in recent years [1]. Most ruptures occur in recreational athletes during sports due to uncoordinated or forceful muscle contraction. Nevertheless, according to degenerative theory, inadequate blood supply of the Achilles tendon causes degeneration, particularly affecting a tendon segment of 2-6 cm proximal to its insertion. Consequently, especially in older individuals, degeneration increases the risk of ruptures, even with minimal force applied to the Achilles tendon [2]. Common risk factors for AATR include increased age, male gender, pharmaceutical agents such as oral and injectable corticosteroids as well as fluoroquinolones, pre-existing Achilles tendinopathy, genetic predisposition, and the presence of systemic disorders, such as hyperlipidemia, arteriosclerosis, renal insufficiency, and inflammatory or autoimmune diseases [2,3].

AATR management is a matter of controversy and may be approached both operatively and nonoperatively [4-6]. Moreover, many surgical techniques have been proposed, roughly categorized into open and minimally invasive repair. However, there is still no agreement regarding the optimal treatment. Operative management is considered to reduce re-rupture risk and lead to better functional outcomes and increased return to sport rates [7,8]. On the other hand, nonoperative management avoids surgical complications, thus being the safest option for low-demand patients [4-7,9]. Regarding operative management, the American Academy of Orthopedic Surgeons (AAOS) recommends a cautious approach in patients with comorbidities, such as diabetes, neuropathy, peripheral vascular disease, immunosuppression, obesity, history of smoking or sedentary lifestyle, and dermatologic diseases [10].

Endoscopic flexor hallucis longus (FHL) tendon transfer is an acceptable surgical option for patients with neglected Achilles tendon rupture, even with comorbidities [11,12]. This technique was first described as a treatment modality for patients with AATR in 2020 [13]. Furthermore, it has also been characterized as a reliable management option for AATR in elite athletes [14]. Novel data also suggest that endoscopic FHL transfer has a favorable cost-effectiveness profile, while nonoperative management demonstrates the highest cost for treating complications [15]. The purpose of this case report is to present a high-risk patient with AATR treated with endoscopic FHL transfer in our department. We aim to emphasize that this minimally invasive operative option may be an acceptable alternative to open techniques or nonoperative management, providing satisfactory functional outcomes without major surgical trauma.

## Case presentation

**Figure 1:**
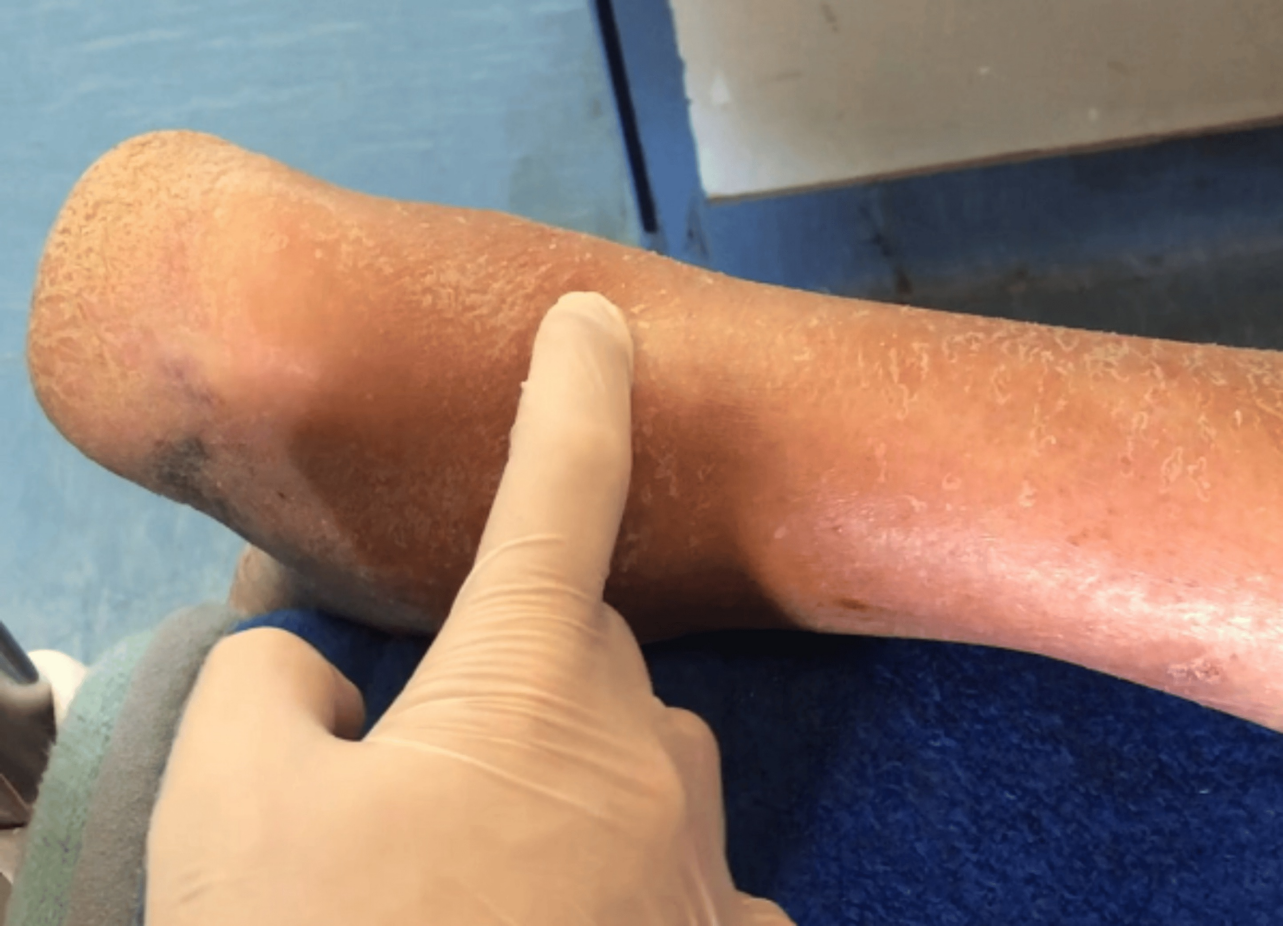
Preoperative evaluation. The examiner’s index finger shows the palpable gap between the Achilles tendon stumps after an acute Achilles tendon rupture. The examiner may also notice a hematoma due to the recent injury, the hyperkeratosis of the heel as well as the cracking and flaking of the calf skin.

**Figure 2:**
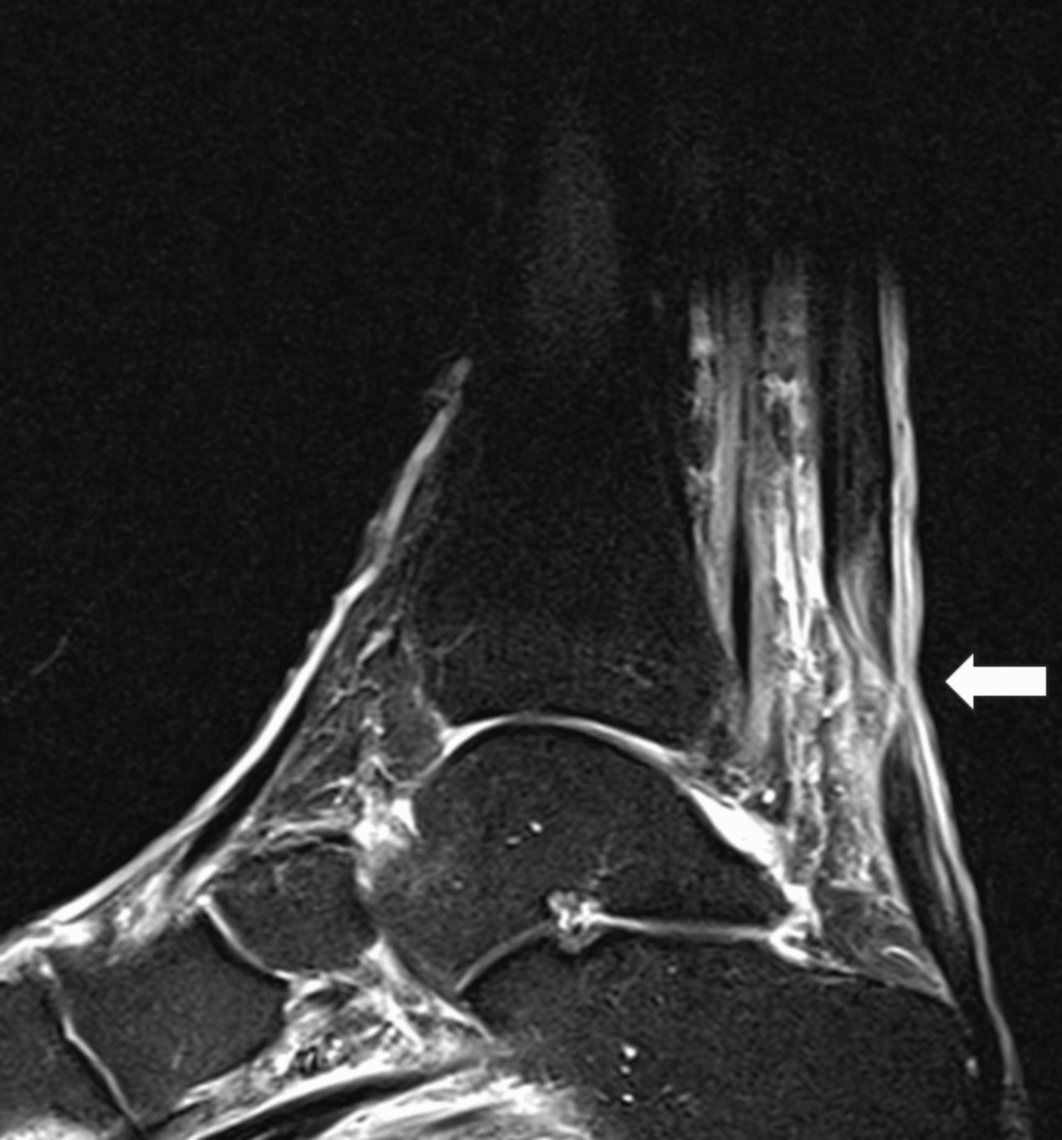
Achilles tendon rupture diagnosis is confirmed with an ankle MRI. Achilles tendon rupture is noted 4.4 cm proximal to the calcaneal insertion (arrow). The tendon stumps present minimal retraction. Increased intratendinous signal is indicative of some degree of Achilles noninsertional tendinopathy in T2 sequence. MRI: Magnetic Resonance Imaging

A 66-year-old male patient with AATR was referred to our department (Figures 1, 2). His tendon was reportedly injured two weeks ago after a misstep of his left foot during aggressive rehabilitation, which he was undergoing following a recent stroke. In addition, the patient’s medical history included long-term treatment of pemphigus with oral methylprednisolone over the previous 20 years, as well as dyslipidemia, hypertension, prediabetes, and smoking. The patient's BMI was 27. No previous surgical history was noted. The patient also reported recent pneumonia treated with levofloxacin and budesonide. Conservative management was initially suggested after considering these comorbidities. However, the patient desired a better functional outcome to resume his stroke rehabilitation regime in full.

Consequently, the option of endoscopic FHL tendon transfer was suggested, and the patient agreed. After providing written informed consent, the patient was operated 20 days post-injury.

**Figure 3:**
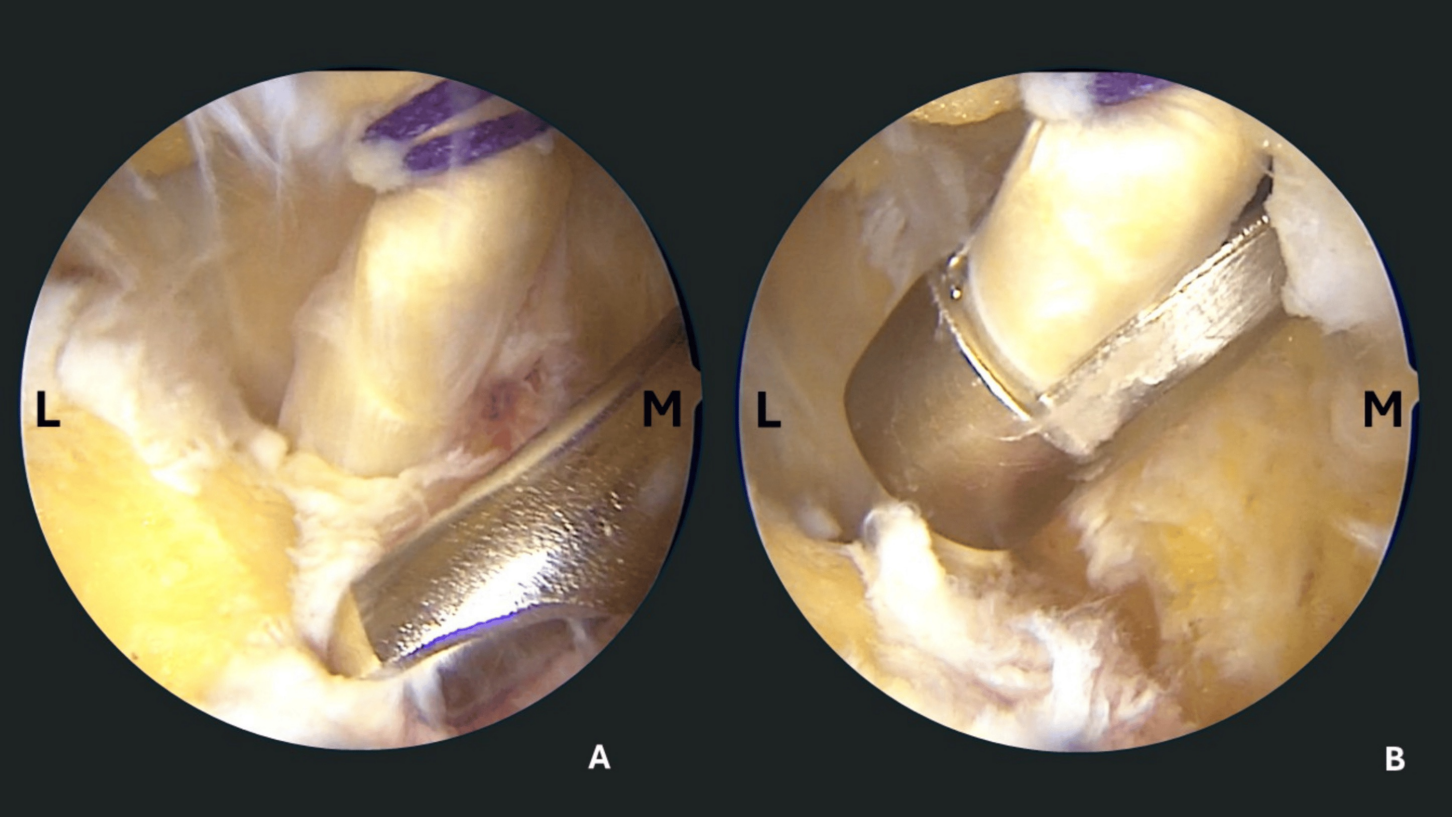
Intraoperative view of FHL harvesting. (A) A towing suture (Vicryl) applies tension to the tendon and the soft tissue around it is debrided with the shaver. (B) A hamstring tendon stripper is introduced through the medial portal and embraces the FHL tendon. Then, it is forwarded into the fibro-osseous sheath to harvest 20mm of tendon length. Caution is required to avoid forceful thrusting of the stripper into the canal due to the risk of damaging the master knot of Henry and the medial plantar nerve. L: Lateral, M: Medial, FHL: Flexor Hallucis Longus

Operative endoscopic FHL tendon transfer was undertaken with the following technique [11-13,16-18]. The patient, under regional anesthesia, was placed in prone position, a tourniquet was applied at the ipsilateral thigh and inflated at 300 mmHg. After proper disinfecting and draping, two portals were established on each side of the Achilles tendon at the level of the tip of the lateral malleolus, a typical posteromedial and a posterolateral portal, according to Van Dijk [18]. A standard 4.0 mm 30^O^ knee scope was inserted through the posterolateral portal, mainly used as a viewing portal in hindfoot endoscopy. A 4.0 mm shaver was introduced through the posteromedial portal to facilitate soft tissue debridement and the recognition of critical anatomical structures [16]. Debridement was limited to the lateral aspect of the FHL to avoid injury of the tibial nerve and the posterior tibial artery. The FHL tendon fascia was released as proximal as possible, to increase approximation between the ruptured Achilles tendon and the FHL muscle belly [11,12]. Then, tension was applied to the FHL tendon through a towing Vicryl suture, and the tendon was harvested with a typical hamstring tendon stripper (Figures 3A, 3B). A strong loop suture was applied to the FHL tendon stump using the Krakow technique [16]. Under fluoroscopy, a calcaneal tunnel was retrospectively drilled as posterior and medial as possible to ensure optimal biomechanical advantage [12,16,19]. The FHL tendon was tensioned and fixated to the calcaneus with a bioabsorbable interference screw (length 25mm, diameter 8mm), while the ankle was positioned at 20 degrees of plantarflexion. Direct Achilles tendon endoscopy was then performed, and the rupture site was debrided with the shaver, aiming to increase bleeding and healing potential [16]. After wound closure, a below-knee cast in equinus position was applied. After two weeks, the cast was exchanged for a walking boot in a neutral position, and partial weight bearing was suggested. Full weight bearing was allowed postoperatively after the fourth week. Physiotherapy sessions were offered after the cast removal and were focused on early ankle motion.

**Figure 4:**
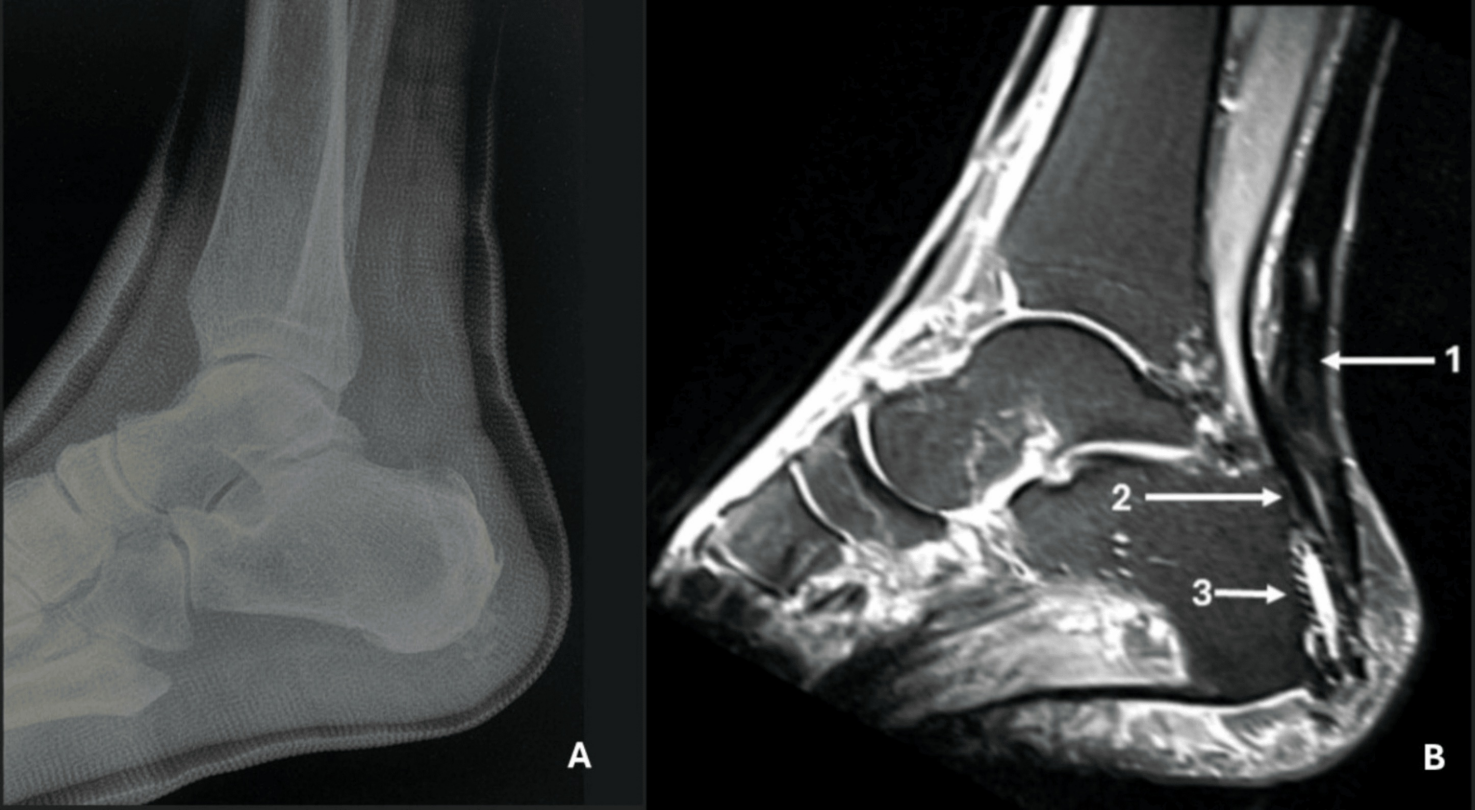
(A) Postoperative lateral ankle radiograph. (B) Postoperative T2 MRI at 24-month follow-up. Arrow 1 indicates the homogenous signal of the healed Achilles tendon. Arrow 2 depicts the transferred FHL tendon. Arrow 3 shows the fixation of the FHL with the bioabsorbable interference screw. MRI: Magnetic Resonance Imaging, FHL: Flexor Hallucis Longus

The patient underwent follow-up examinations at six weeks, 12 weeks, 12, and 24 months (Figures 4A, 4B).

No complications were recorded at the 24-month follow-up. The patient was satisfied regarding the operated limb, as he reported being able to walk the same distance compared to the one before the rupture. The importance of walking is highlighted, as it enables him to maintain an adequate fitness level, vital for effectively managing his cardiovascular comorbidities. He could also continue his strengthening rehabilitation exercises without any additional exertion. Patient satisfaction was evaluated at 12 months using the Achilles tendon total rupture score (ATRS), which has been assessed for its validation and cross-cultural adaptation to the patient’s native language [20,21]. The patient scored 92 out of a maximum of 100 points. Additional functional outcome measures included measurements of ankle and calf circumference, ankle passive and active ankle range of motion (ROM), and hallux flexion dynamometry. The results regarding the injured limb were compared to those of the healthy contralateral side. These results are shown in Table 1.

**Table 1:** Functional outcome measures ROM: Range of Motion, N: Newton

Functional outcome measure	Right leg	Left leg (operated)	Difference
Calf circumference	32.5 cm	32 cm	- 0.5 cm
Ankle circumference	22 cm	22 cm	0
Hallux flexion power	133 N	69 N	- 48%
Active ROM	51^o^	45^o^	- 6^o^
Passive ROM	57^o^	48^o^	- 9^o^

## Discussion

AATR management is still debatable and there is no consensus regarding which patients would benefit more from operative management. However, AAOS in 2010 provided recommendations about which patient categories should be approached with skepticism regarding operative management [10]. The best option depends on each patient’s characteristics, lifestyle, and demands, as well as the surgeon’s preference and experience. Although novel data support that the combination of minimally invasive surgery followed by early rehabilitation protocol seems to be the best treatment strategy [5,22], an individualized approach may sometimes be necessary to meet the patient's needs. This case report demonstrates that there is another acceptable therapeutic approach for high-risk patients with AATR. Our patient scored a satisfactory 92 out of a maximum of 100 points in ATRS, even though the ipsilateral foot had a strength deficit. This hallux flexion power deficit is mainly attributed to the recent stroke, rather than the FHL transfer per se since the FHL tendon was harvested proximal to the master knot of Henry [16]. It is reported that the hallux flexion power deficit in some cases does not appear to have functional implications [11-13]. The remaining functional outcome parameters, such as calf-ankle circumference, and active-passive ROM, did not demonstrate clinically significant differences. Moreover, it is unclear whether the recent stroke affected these outcomes.

Tendon elongation is a common complication of AATR and may occur at the rehabilitation stage regardless of the chosen treatment, leading to an inferior outcome [23]. Achilles tendon elongation affects foot and ankle biomechanics, plantar flexion strength, maximum calf circumference, and muscular volume [9]. However, despite this mechanical disadvantage, Achilles tendon elongation does not necessarily result in detrimental effects regarding Patient Reported Outcome Measures [9]. It is supported that augmentation of percutaneous Achilles tendon repair with FHL transfer reduces subsequent elongation and yields better functional outcomes. Nevertheless, in this study, patients aged over 50 or with comorbidities have been excluded [24]. A possible explanation is that the FHL tendon functions as an internal brace of the injured foot, therefore minimizing the retraction of the torn Achilles tendon stumps [16].

The FHL tendon transfer presents some advantages compared to other tendon transfers [25]. FHL muscle is more robust than nearby muscles and its force vector resembles the vector produced by the gastro-soleus-Achilles tendon complex [13,16,25]. Moreover, it fires “in phase” with the complex, resulting in normal ankle muscle balance [16,25]. This muscle also demonstrates a 5% compensatory hypertrophy in case of ipsilateral Achilles tendon rupture [26]. From a technical point of view, FHL tendon harvesting is relatively easy and has sufficient length and diameter to ensure proper fixation to the calcaneus [27]. Optimal tunnel establishment and uncomplicated tendon fixation yield superior biomechanical advantages [16]. The proximity of the well-vascularized FHL muscle belly to the Achilles tendon enhances its vascularity, increasing the Achilles tendon healing potential [13,25]. This proximity also decreases surgical manipulations, leading to fewer iatrogenic neurovascular complications [12,13,25].

A complication rate of 14.8% is reported in open, mini-open, and endoscopic FHL tendon transfers for chronic Achilles tendon ruptures [25]. Higher complication rates, up to 52%, are reported after open FHL tendon transfers, whereas infections and wound healing complications are reported in 34% [28]. Moreover, open Achilles tendon repair demonstrates a considerably high wound complication rate in patients with risk factors compared to the ones without (42.1 vs 6.2%) [29]. Endoscopic and minimally invasive approaches have gained popularity in recent years, minimizing soft tissue trauma. Therefore, endoscopic FHL transfer could be a safer treatment choice for high-risk patients. Vernois et al. reported no complications and satisfactory outcomes in eight high-risk patients with neglected Achilles tendon rupture [11]. Their comorbidities included obesity, diabetes, renal impairment, corticosteroid usage, and post-thrombotic limb, and all patients were older than 65 years. Vega et al. also reported no complications in 22 patients with chronic noninsertional rupture, although patients were not considered high-risk [12]. Two high-risk patients with chronic ruptures underwent endoscopic FHL transfer in a case series of Lee with acceptable outcomes [30]. Batista et al. reported 56 male patients with AATR, of which 32.1% had at least one risk factor. This group of patients demonstrated a 3.5% infection rate and an overall complication rate of 17.8% [13]. Abdelatif et al. reported a case series of 27 elite athletes with AATR and an overall complication rate of 7% (3.5% infections). This group, however, comprised fewer patients with at least one risk factor (18.5%) [17].

Ultimately, this case report emphasizes that there is a safe alternative surgical treatment option for high-risk patients with AATR. Large case series and randomized controlled trials comparing this treatment modality with conservative management and other surgical techniques are necessary to provide valuable insights regarding the optimal treatment option for these individuals.

## Conclusions

High-risk patients with AATR pose a therapeutic challenge since they are not the best candidates for surgical management. Conservative treatment is a widely adopted safe option for these patients, as it avoids devastating surgical complications, such as wound dehiscence or infection. Endoscopic FHL transfer has gained popularity as a treatment modality for patients with neglected Achilles tendon ruptures. Still, it has recently been characterized as a reliable choice for patients with acute ruptures. High-risk patients with AATR may benefit from the broader adoption of minimally invasive techniques, such as endoscopic FHL transfer.
